# The Influence of Universal Health Coverage on Life Expectancy at Birth (LEAB) and Healthy Life Expectancy (HALE): A Multi-Country Cross-Sectional Study

**DOI:** 10.3389/fphar.2018.00960

**Published:** 2018-09-18

**Authors:** Chhabi L. Ranabhat, Joel Atkinson, Myung-Bae Park, Chun-Bae Kim, Mihajlo Jakovljevic

**Affiliations:** ^1^Institute for Poverty Alleviation and International Development, Yonsei University, Wonju, South Korea; ^2^Health Science Foundations and Study Center, Kathmandu, Nepal; ^3^Manmohan Memorial Institute for Health Sciences, Kathmandu, Nepal; ^4^Graduate School of International and Area Studies (GSIAS), Hankuk University of Foreign Studies (HUFS), Seoul, South Korea; ^5^Department of Gerontal Health and Welfare, Pai Chai University, Daejeon, South Korea; ^6^Department of Preventive Medicine, Yonsei University Wonju College of Medicine, Wonju, South Korea; ^7^Faculty of Medical Sciences, Global Health Economics & Policy PhD Curriculum, University of Kragujevac, Kragujevac, Serbia

**Keywords:** universal health coverage, multi-country, life expectancy at birth, healthy life expectancy, record linkage

## Abstract

**Background:** There are substantial differences in long term health outcomes across countries, particularly in terms of both life expectancy at birth (LEAB) and healthy life expectancy (HALE). Socio-economic status, disease prevention approaches, life style and health financing systems all influence long-term health goals such as life expectancy. Within this context, universal health coverage (UHC) is expected to influence life expectancy as a comprehensive health policy. The aim of the study is to investigate this relationship between Universal Health Coverage (UHC) and life expectancy.

**Method:** A multi-country cross-sectional study was performed drawing on different sources of data (World Health Organization, UNDP-Education and World Bank) from 193 UN member countries, applying administrative record linkage theory. Descriptive statistics, *t*-tests, Pearson correlations, hierarchical linear regressions were utilized as appropriate.

**Result:** Global average healthy life years was shown to be 61.34 ± 8.40 and life expectancy at birth was 70.00 ± 9.3. Standardized coefficients from regression analysis found UHC (0.34), child vaccination (Diphtheria Pertussis Tetanus−3: 0.17) and sanitation coverage (0.31) were associated with significantly increased life expectancy at birth. In contrast, population growth was associated with a decrease (0.29). Likewise, unit increases in child vaccination (DPT 3), sanitation and UHC would increase healthy life expectancy considerably (0.18, 0.31, and 0.40 respectively), whereas the same for population growth reduces healthy life expectancy by 0.28.

**Conclusion:** Universal Health Coverage (UHC) is a comprehensive health system approach that facilitates a wide range of health services and significantly improves the life expectancy at birth and healthy life expectancy. This study suggests that specific programs to achieve UHC should be considered for countries that have not seen sufficient gains in life expectancy as part of the wider push to achieve the Sustainable Development Goal (SDG).

## Introduction

There have been significant gains in life expectancy worldwide, with global life expectancy at birth (LEAB) increasing by 6 years (65–71) since 1990 (World Health Organization, 2014a). However, there is profound disparity in life expectancy between countries and regions (Moser et al., 2005; Vågerö, 2014). Life expectancy at birth for low-income countries is now 62 years, compared to 79 years in high-income countries, and there is a 34 year gap between the lowest country (Lesotho, 50 years) and the highest (Japan, 84 years) (World Health Organization, 2014a). The global variation in healthy life expectancy (HALE), which gauges the status of morbidity and mortality as part of population health (Guest et al., 2006), follows a similar pattern to LEAB.

Many explanatory factors such as per capita income, education, government and private health expenditure, access to safe water, physician ratio, nutritional outcomes, geographical status and urbanization have been found to significantly influence life expectancy (Jakovljevic et al., 2016a), especially in developing countries (Husain, 2002; Kabir, 2008). Higher mortality (infant, child and adult) directly reduces life expectancy. Socio-economic status, disease control approaches, life style and existing health care financing systems are also related to mortality and morbidity (Beltrán-Sánchez and Soneji, 2011; Dieleman et al., 2017; Ranabhat et al., 2017a). Among these factors, both LEAB and HALE are strongly influenced by health interventions and other aspects of social development (Chan and Kamala Devi, 2015). A major reason for the variation between countries is that life expectancy reflects the mortality pattern of a population as well as the overall impact of the health system (Jakovljevic et al., 2017a). An effective health system and policy are able to balance key predictors for both types of life expectancies, such as socio-economic factors and lifestyle patterns (Kabir, 2008; Lin et al., 2012). So, LEAB and HALE reflect the accessibility, quality and overall coverage of a comprehensive range of health care services (Olshansky et al., 2005).

As such, the role of Universal Health Coverage (UHC), which ensures broad population access to promotive, preventive, curative and rehabilitative health services^1^, is receiving growing attention. UHC is defined as a combination of a legal assurance of health insurance, >90% coverage of health insurance and skilled birth attendance (Stuckler et al., 2010). The first example of universal health coverage that delivered access to basic medical services even to the poorest citizens, was the Soviet Union's famous Semashko system that was established in the early 1930s (Semashko, 1934; McMichael, 1942). The World Health Organization (WHO) has called for all countries to adopt UHC (Carrin et al., 2005). Achieving universal health coverage has also been included as a target in the newly adopted Sustainable Development Goals (SDGs). However, to date, there has been inadequate investigation of its effect on long-term health outcomes (Rancic and Jakovljevic, 2016). There have been some country studies, such as Davis and Huang (2008), who found life expectancy and other health outcomes significantly increased in Taiwan after the adoption of UHC (Davis and Huang, 2008). However, the association between universal health coverage and life expectancy, and strength of the association has not been systematically tested comparatively.

We examine the influences of UHC, utilizing the largest feasible sample of countries. We also address important components such as socioeconomic status, disease prevention coverage, prevalence of youth tobacco use (Olshansky et al., 2005) and alcohol consumption (Ranabhat et al., 2018a) and health financing factors (Jakovljevic et al., 2017b). Moreover, the weight of UHC would be tested on other long-term health outcomes and its importance could be highlighted in order to reduce health inequality within the countries and across the countries. So, the aim of the study is to examine the relation between Universal Health Coverage (UHC) and life expectancy.

## Materials and methods

This is a multi-country cross-sectional study of all UN member countries for the period 2010/12.

### Research hypothesis model

**Figure 1:**
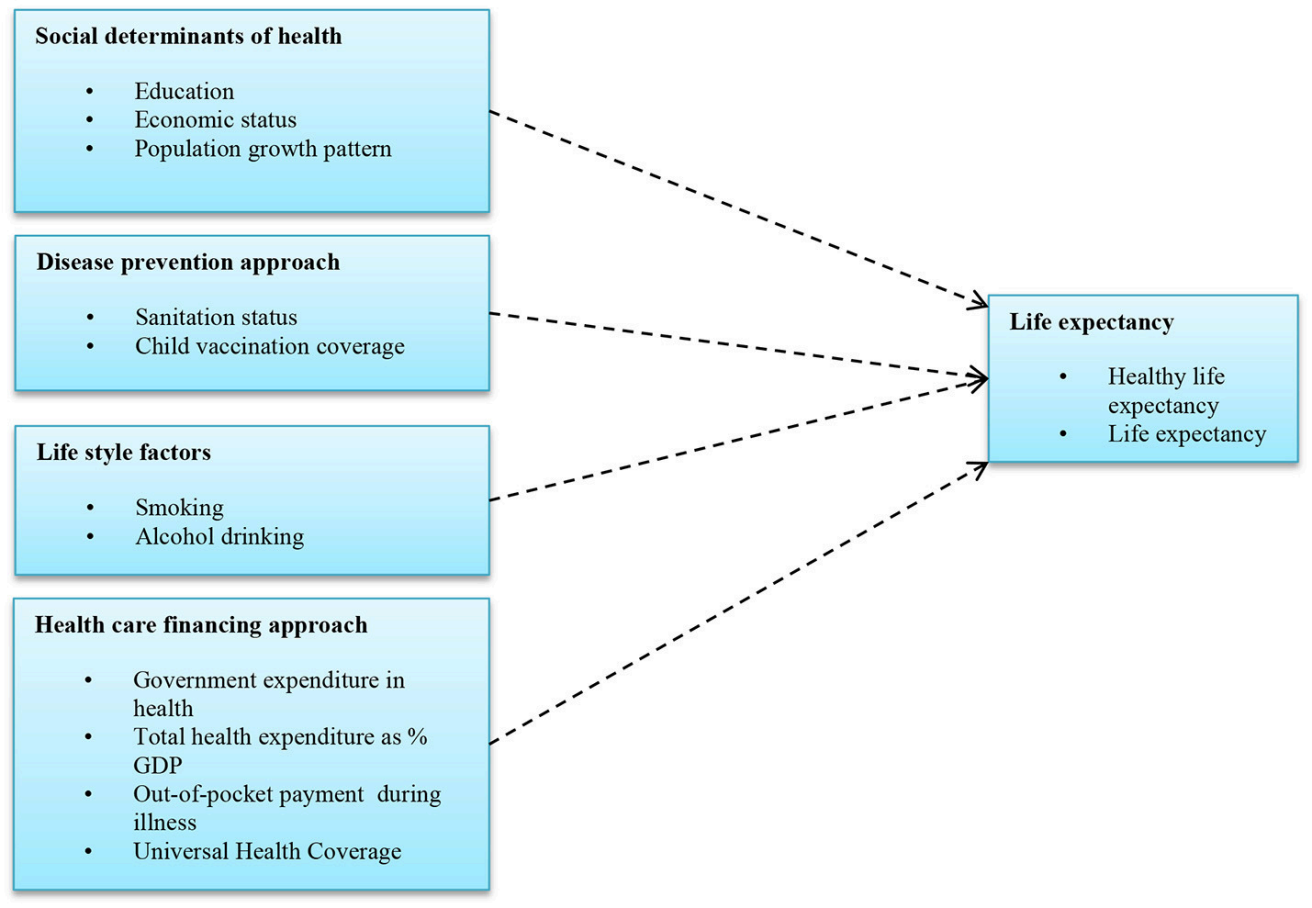
Conceptual model influencing life expectancy with different predictors.

Previous research explored different factors affecting health outcomes such as life expectancy. Mondal and Shitan (2012) observed the association between socio-economic and demographic factors and life expectancy (Mondal and Shitan, 2012). Likewise, Paasche-Orlow and Wolf (2007) examined the connection with health literacy, finding a positive effect on life expectancy via increased health coverage (Paasche-Orlow and Wolf, 2007). Studies by the WHO (World Health Organization, 2014a) and Moreno-Serra and Smith (2012) indicated that there is an association between universal health insurance and life expectancy (Moreno-Serra and Smith, 2012). Edwards (2011) further clarified that UHC is a comprehensive health policy and assures all types of health care service without financial deficiency (O'Connell et al., 2014). Socio-demographic, disease prevention indicators, life style and health financing components have been found to have some association with life expectancy in separate studies (Jakovljevic et al., 2016b). We updated these models and compiled the necessary components with life expectancies using multiple sources of data. The research hypothesis model based on these previous studies is shown in Figure 1.

### Data base model

Data were compiled based on Record Linkage theory applied by (Dunn, 1946). This approach aims to create new statistics through combining verified data that may have been collected for different purposes. Jutte (2011) applied and refined administrative record linkage as a tool for public health research (Jutte et al., 2011). Our database is derived from combined health data from the World Bank^2^, youth smoking prevalence and alcohol consumption rates from the WHO (World Health Organization, 2010, 2014a), universal health care coverage from Stuckler et al. (2010), and education status from the UNDP^3^ Those data are publicly accessible from the respective organizations via their websites.

### Data management

From these different sources, we created new data by country for all 193 UN members. Data and outliers were cross-checked and missing data from some countries were excluded from the analysis. After verification and re-verification, the data were exported for analysis with the Statistical Package for the Social Sciences (SPSS)-20 (SPSS I, 2011).

### Data analysis model

First, global average results were presented in means and standard deviations. In the second phase, Pearson correlation coefficients were computed. In the third step, multivariate analysis was performed using a hierarchical linear regression model. The data was interpreted by standard beta coefficients and *p*-values at the significance level of < 0.05.

### Selection of variables and description

The independent variables were selected according to the research model, country-wise data availability and policy implication.

The standard definitions of all dependent and independent variables are described below (Table 1).

**Table 1:** Description of variables and data sources.

**Variables**	**Source**
**INDEPENDENT VARIABLES**	
**Combined gross enrollment (CGE)** rate (both sexes) is the number of students enrolled in primary, secondary and tertiary levels of education, regardless of age, as a percentage of the population of theoretical school age for the three levels. It is a comprehensive indicator of education.	Human Development Report combined gross enrollment- 2012^3^
**Population growth rate (PGR)** is the increase in a country's population during a period of time, usually one year, expressed as a percentage of the population at the start of that period. It reflects the number of births and deaths during the period and the number of people migrating to and from a country.	World Bank data base on health 2012^2^
**Economic growth rate (EGR)** is the sum of average gross value added by all resident producers in the economy plus any product taxes and minus any subsidies not included in the value of the products from 2006 to 2010 in percentage terms.	World Bank database on health 2012^2^
**Access to improved sanitation** refers to the percentage of the population using improved sanitation facilities. Improved sanitation facilities are likely to ensure hygienic separation of human excreta from human contact. They include flush/pour flush (to piped sewer system, septic tank, and pit latrine), ventilated improved pit (VIP) latrine, pit latrine with slab, and composting toilet.	World Bank database on health 2012^2^
**Child immunization** measures the percentage of children ages 12–23 months who received vaccinations before 12 months or at any time before the survey. A child is considered adequately immunized against Diphtheria, Pertussis (whooping cough), and Tetanus (DPT) after receiving three doses of vaccine.	World Bank database on health 2012^2^
**Youth tobacco prevalence** is the mean percentage of boys and girls among that group who smoke any form of tobacco, including cigarettes, cigars, pipes or any other smoked tobacco products. Data includes daily and non-daily or occasional smoking.	WHO report on global tobacco epidemic–2013 (World Health Organization, 2014a)
**Alcohol consumption** ratio is the mean consumption of alcohol in liters by adult males and females within one year.	Global information system on alcohol and health 2010 (World Health Organization, 2010)
**Out-of-pocket expenditure** is any direct outlay by households, including gratuities and in-kind payments, to health practitioners and suppliers of pharmaceuticals, therapeutic appliances, and other goods and services whose primary intent is to contribute to the restoration or enhancement of the health status of individuals or population groups. It is a part of private health expenditure.	World Bank database on health 2012^2^
**Total health expenditure** is the sum of public and private health expenditure. It covers the provision of health services (preventive and curative), family planning activities, nutrition activities, and emergency aid designated for health but does not include provision of water and sanitation.	World Bank database on health 2012^2^
**Public (Government) health expenditure** consists of recurrent and capital spending from government (central and local) budgets, external borrowings and grants (including donations from international agencies and nongovernmental organizations), and social (or compulsory) health insurance funds.	World Bank database on health 2012^2^
**Universal health care (coverage)** means that all people can use the promotive, preventive, curative, rehabilitative and palliative health services they need, of sufficient quality to be effective, while also ensuring that the use of these services does not expose the user to financial hardship. More specifically, it is defined as legislation mandating Universal Health Care, along with >90% health insurance coverage and >90% skilled birth attendance.	Stuckler et al. (2010)
**DEPENDENT VARIABLES**	
**Life expectancy at birth** indicates the number of years a newborn infant would live if prevailing patterns of mortality at the time of its birth were to stay the same throughout its life.	World Bank data base on health 2013^2^
**Healthy life expectancy** measures the number of remaining years that a person of a certain age is expected to live without disability and it is actually a disability-free life expectancy.	Salomon et al. (2013)

### Structure of variables

All of the above variables are numerical except universal health coverage. Principally, UHC is a combination of 3 indicators: legal obligation of universal health insurance, skilled birth attendance rate and health insurance coverage. Following Stuckler et al. (2010) we applied UHC (yes) = 1 to countries that had achieved these three indicators and UHC (no) = 0 to countries that had not. After making consistent variables, we performed the linear regression models.

### Validity and reliability

There are very few questions about the validity and reliability of Word Bank, WHO and UNDP data used in this study. After data entry, we performed more than two rounds of crosschecking to assure the quality. Likewise, the normality of the data was verified by the observation of histograms and consistency of data was checked by Cronbach's alpha in appropriate variables. As UHC is a composite definition, we checked for multicollinearity with other variables. Based on the coefficient output, collinearity statistics obtained a variance inflation factors (VIF) value between 1 and 10, indicating that multicollinearity was not an issue. The ethical approval is not applicable because this study based on secondary sources of data available for open access.

### Method of observation

An association with outcome “Y (Y_1_, Y_2_) = β_1_x_1_ +β_2_x_2_ +β_3_x_3_ + β_4_x_4_ …….…. β_n_x_n+_ α

I.e., Outcomes (Y = Y_1_, Y_2_) i.e., healthy life expectancy (HALE) and life expectancy at birth (LEAB) = “β_1_ Socio-demographic variables + β_2_ disease prevention variables + β_3_ life style variables + β_4_ Health financing variables” + α.

## Result

Table 2 shows the mean values for all dependent and independent variables. The results reveal that 57 countries out of 194 (29.38%) have already achieved universal health coverage (UHC), and the global average for healthy life expectancy is 61.34 ± 8.40 and life expectancy at birth is 70.00 ± 9.32 in years. There is a 31 year healthy life expectancy gap between the highest ranked country (Singapore, 75 years) which has achieved UHC and the lowest ranked country (Sierra Leone, 44 years) which has yet to achieve UHC. Similarly, there is 35-year gap in life expectancy at birth between the highest LEAB country with UHC (Japan 83 years), and the lowest that has yet to achieve UHC (also Sierra Leone 48 years). On average, life expectancy at birth and healthy life expectancy was significantly higher (*p* < 0.001) in countries that have achieved UHC (HALE 68.92 ± 4.04 and LEAB 78.07 ± 4.13) than in countries that are yet to achieve UHC (HALE 58.23 ± 7.66 and LEAB 66.77 ± 8.59).

**Table 2:** Descriptive results.

**Variables (in years)**	**Number of countries**	**Mean ± Std. deviation**
Combined gross enrollment of education (both sexes)–(2012) %	180	95.80 ± 31.08
GDP growth rate by country–(2008-2012) %	185	3.16 ± 3.17
Population growth rate by country–(2012) %	192	1.41 ± 1.33
Sanitation coverage by country in %–(2012)	188	70.59 ± 23.79
Child vaccine coverage (DPT 3) by country–(2012) %	190	89.23 ± 13.05
Adult alcohol consumption /year liter by country–(2010) in liter	193	5.72 ± 4.30
Prevalence of youth tobacco use by country–(2011) %	132	22.07 ± 9.27
Total health expenditure as percent of GDP–(2012) %	183	6.80 ± 2.94
Out of Pocket Payment (OPP) among total expenditure–(2012)%	185	32.08 ± 18.97
Government expenditure in Health in–(2012)%	186	11.63 ± 4.90
Life expectancy at birth by countries–(2012) years	184	70.00 ± 9.32
Healthy life years by country–(2012) years	179	61.34 ± 8.40

*Cronbach's alpha, 0.692*.

A raw correlation matrix was observed among all variables. It shows that healthy life years and life expectancy at birth were positively associated (*p* < 0.05) with universal health care and child vaccination (DPT 3) coverage and negatively associated with population growth rate and adult alcohol consumption (Table 3).

**Table 3:** Raw correlation matrix between all observations.

**S.N. Observations**			**2**	**3**	**4**	**5**	**6**	**7**	**8**	**9**	**10**	**11**	**12**	**13**
1	CGE-EDU (012)	1												
2	EGR (06- 010)	−0.140	1											
3	PGR (012)	0.109	−0.494^**^	1										
4	DPT3 (012)	0.046	0.192^*^	−0.335^**^	1									
5	SAN COV (012)	−0.065	0.029	−0.150^*^	0.294^**^	1								
6	ALCOHOL CON	−0.213^*^	−0.282^**^	0.349^**^	−0.146	−0.021	1							
7	YOUTH_TOB_P	−0.307^**^	0.006	−0.176	−0.119	0.018	0.327^**^	1						
8	UHC	0.003	0.337^**^	−0.272^**^	0.292^**^	0.147^*^	−0.241^**^	−0.003	1					
9	OPP (012)	−0.074	−0.381^**^	−0.158^*^	−0.214^**^	0.053	−0.204^**^	0.157	−0.202^**^	1				
10	THE (012)	0.028	0.071	−0.051	0.030	0.011	−0.051	−0.341^**^	−0.098	0.116	1			
11	GHE (012)	0.003	0.245^**^	−0.273^**^	0.205^**^	0.101	−0.128	−0.190^*^	0.240^**^	−0.598^**^	−0.101	1		
12	Healthy life years (012)	0.006	0.301^**^	−0.487^**^	0.564^**^	0.364^**^	−0.300^**^	−0.086	0.583^**^	−0.177^*^	0.071	0.286^**^	1	
13	Life expectancy (012)	0.041	0.254^**^	−0.478^**^	0.533^**^	0.331^**^	−0.291^**^	0.047	0.540^**^	−0.196^**^	−0.077	0.247^**^	0.966^**^	1

* *Correlation is significant at the 0.05 level (2-tailed)*.

** *Correlation is significant at the 0.01 level (2-tailed). CGE-EDU, Combine gross enrollment rate in education; EGR, Economic/GDP growth rate; PGR, Population growth rate; DPT, Diphtheria, Pertussis, Tetanus; SAN COV, Sanitation coverage; ALCOHOL CON, Alcohol consumption; YOUTH_TOB_P, Youth tobacco prevalence; THE, Total health expenditure; OPP, Out-of-pocket payment; GHE, Government health expenditure and UHC, Universal health coverage (yes/no)*.

The following box (Box 1) shows the multicollinearity status of independent variables with universal health coverage (UHC) because many independent variables would relate to it. As shown, the variance inflation factors (VIF) were less than 3 and well acceptable in terms of collinearity effect.

Box 1 **Status of multicollinearity between independent variables with universal health coverage (UHC).**

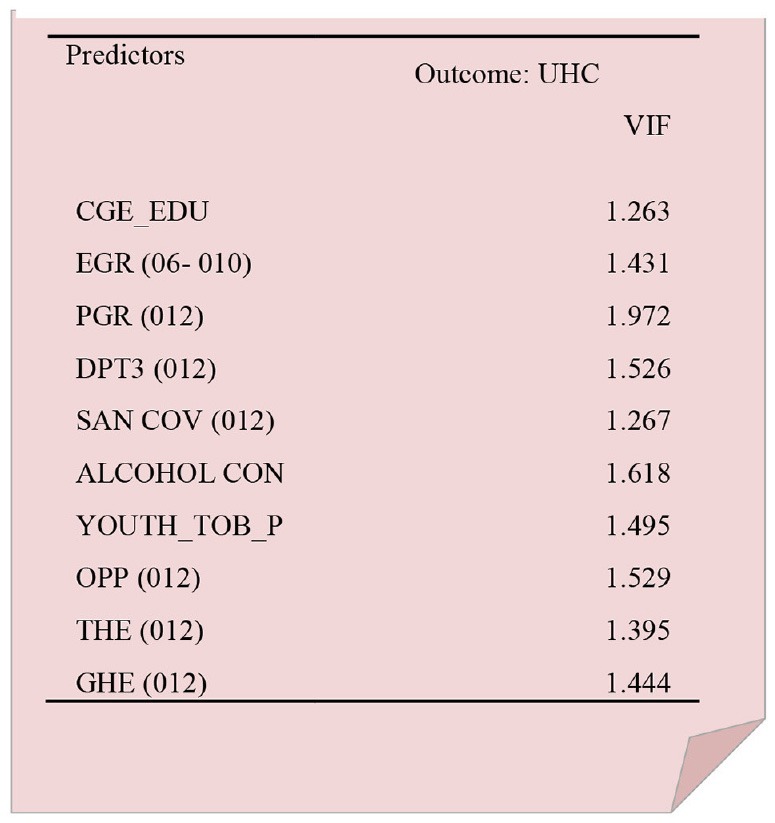

Table 4 shows the association of life expectancy at birth with predictors. The predictors related to social determinants, disease prevention, health behavior and health financing with UHC and life expectancy as outcome for the four models. According to our regression equation model, UHC, sanitation coverage and child vaccination (DPT 3) were associated with significantly increased life expectancy at birth by 0.34, 0.31, and 0.17 (*p* < 0.05). Likewise, population growth is associated with significantly reduce LEAB by 0.29 (*p* < 0.05). For each model, UHC had the highest beta coefficient value among the independent variables in the equation.

**Table 4:** Regression analysis between predictors and life expectancy at birth.

**Independent variables**	**Dependent variable (life expectancy at birth)**							
	**Model 1**		**Model 2**		**Model 3**		**Model 4**	
	**Std coff**.	***P*****-value**	**Std coff**.	***P*****-value**	**Std coff**.	***P*****-value**	**Std coff**.	***P*****-value**
(Constant)	73.9	<0.001	54.5	<0.001	58.6	<0.001	57.2	<0.001
**SOCIAL DETERMINANTS OF HEALTH**								
Combined gross enrollment of education in all age %	0.110	0.107	0.028	0.198	0.038	0.697	0.04	0.571
Economic growth rate %	0.001	0.996	−0.023	0.899	0.028	0.791	0.07	0.082
Population growth rate %	−0.381	<0.001	−0.322	0.001	−0.301	0.003	−0.29	0.021
**ACHIEVED UNIVERSAL HEALTH COVERAGE (YES - REFERENCE)**	0.390	<0.001	0.381	<0.001	0.350	<0.001	0.348	<0.001
**DISEASE PREVENTION**								
Sanitation coverage in %			0.330	<0.001	0.227	0.004	0.311	0.002
DPT-3 vaccine coverage in %			0.176	0.037	0.164	0.114	0.171	0.049
**HEALTH BEHAVIOR**								
Adult alcohol consumption per year per liter					−0.088	0.423	−0.11	0.465
Prevalence of youth smoking in %					−0.046	0.611	−0.01	0.554
**HEALTH FINANCING POLICY**								
Percent of OPP on health among total expenditure							−0.10	0.071
Total health expenditure as percent of GDP							0.02	0.711
Government expenditure in health %							0.07	0.692
R^2^	0.28	0.53	0.57		0.66			
Adjusted R^2^	0.27	0.52	0.54		0.62			
*p-value*	<0.001	<0.001	<0.001		<0.001			

## Discussion

Life expectancy is the average period that an individual may expect to live and healthy life expectancy is the expected years of healthy life within this life expectancy. All age specific deaths—such as neonatal, infant, child, adult mortality and maternal mortality—ultimately reduces life expectancy (Abajobir et al., 2017; Barber et al., 2017). And reducing infant and children mortality is particularly important in raising life expectancy. Life expectancy is influenced by many factors; however, it is not an immediate health outcome. Global average healthy life expectancy and life expectancy at birth were 61 and 70 years respectively, which is the cumulative achievement of health systems and policies after many years. Global LEAB in 1970, 2000, and 2012 were 59, 67, and 70 years (Edwards, 2011). A recent study shows that from 1990 to 2013, LEAB rose by 6.2 (65.3–71.5) years, healthy life years rose by 5.4 (56.9–62.3) (Murray et al., 2015). The application of different data continued to show similar trends. The countries which have achieved UHC have about a 20-year gap, but there is a 34-year gap in average HALE and LEAB for those yet to achieve UHC. This is a huge disparity in life expectancy among those countries (Ranabhat et al., 2017a)^4^ Life expectancy has been increasing in high income countries that achieved universal health care and reduced risk factors through effective health policy (Nolte and McKee, 2004; Di Cesare et al., 2013; Ogura and Jakovljevic, 2014; Mathers et al., 2015). In our findings, UHC has the greatest influence in LEAB and HALE among other predictors. For example, as shown in the standardized coefficient values in Table 5, a one unit increase in UHC results in a 0.40 rise in HALE. The values for sanitation coverage and child vaccine coverage are somewhat lower (0.31 and 0.18, respectively). On the other hand, one unit of population growth reduces HALE by 0.28 in the full model. The other models show a similar pattern.

**Table 5:** Regression analysis between predictors and healthy life expectancy.

**Independent variables**	**Dependent variable (Healthy life expectancy)**							
	**Model 1**		**Model 2**		**Model 3**		**Model 4**	
	**Std coff**.	***P*-value**	**Std coff**.	***P*-value**	**Std coff**.	***P*-value**	**Std coff**.	***P*-value**
(Constant)	64.2	<0.001	36.5	<0.001	36.0	<0.001	45.4	<0.001
**SOCIAL DETERMINANTS OF HEALTH**								
Combined gross enrollment of education in all age %	0.206	0.005	0.183	0.003	0.171	.019	0.04	0.562
Economic growth rate %	0.197	0.003	0.135	0.015	0.103	.100	0.01	0.601
Population growth rate %	−0.331	0.001	−0.255	0.016	−0.161	.058	−0.28	0.002
**ACHIEVED UNIVERSAL HEALTH COVERAGE (YES - REFERENCE)**	0.430	<0.001	0.420	<0.001	0.418	<0.001	0.407	<0.001
**DISEASE PREVENTION**								
Sanitation coverage in %			0.334	<0.001	0.324	<0.001	0.311	0.001
DPT-3 vaccine coverage in %			0.220	0.006	0.230	.000	0.182	0.041
**HEALTH BEHAVIOR**								
Adult alcohol consumption per year per liter					0.090	0.225	−0.10	0.420
Prevalence of youth smoking in %					0.021	0.785	−0.02	0.574
**HEALTH FINANCING POLICY**								
Percent of OPP on health among total expenditure							−0.18	0.045
Total health expenditure as percent of GDP							0.01	.617
Government expenditure in health %							0.14	.081
R^2^	0.32	0.54	0.58		0.68			
Adjusted R^2^	0.28	0.51	0.56		0.64			
*p-value*	<0.001	<0.001	<0.001		<0.001			

Behind UHC, population growth, sanitation coverage, vaccination coverage, and out-of-pocket expenditure all influenced LEAB and HALE (Ranabhat et al., 2018b). Bloom summarized that vaccination and sanitation coverage decreases morbidity and mortality and increases economic growth (Bloom, 2011). Examples include the human papillomavirus virus vaccine for adolescents in Peru (Goldie et al., 2012) and rotavirus vaccine in India that have reduced mortality and increased life expectancy (Esposito et al., 2011). This is similar to our study, but we failed to find a direct impact for DPT-3 on life expectancy. Out-of-pocket payments deepen the poverty headcount (Tomini et al., 2013) and reduce healthy life years and life expectancy, consistent with our results (Jakovljevic and Getzen, 2016). Among all risk factors, unimproved water and sanitation accounted for 6.8% of disability-adjusted life years (DALYs) in 1990, 3.7% in 2000 and 0.9% in 2010 (Lim et al., 2013). This indicates that public health programs like sanitation and vaccination also affect life expectancy. Generally, a positive economic growth rate should increase health status. However, after UHC achievement, economic growth tended to be faster than usual. For example, after the achievement of UHC in South Korea, the economic growth rate almost doubled (Kwon, 2009). This suggests that economic growth may be a proxy advantage of UHC (Bloom et al., 2004).

Perhaps more useful than a global view is the observation of life expectancy in countries that have and have not yet achieved UHC that are otherwise similar. Salomon compared life expectancy and healthy life expectancy between 1990 and 2010 (Salomon et al., 2013). He found life expectancy and healthy life expectancy trends increased faster for countries with UHC. For example, for the United States, which does not have UHC, life expectancy and healthy life expectancy in 1990 was 72 and 63 years, respectively. After 20 years (2010), it reached 76 and 66 years. This was an increase of only about 3 years. But in the UK, which achieved UHC in the 1950s, the increase was 5 years (LE 73-79 and HALE 63-67). Likewise, two countries yet to achieve UHC, Brazil and China, increased LE and HALE by about 4.5 years. In contrast, South Korea and Ireland (with UHC) (Jakovljevic et al., 2017c), increased their values by more than 6 years in the same time frame. We can also see life expectancy trends in countries before and after achieving UHC^5^. For example, Finland declared UHC in 1972. In the 10 years prior to UHC, LEAB increased by 3 years (from 68 to 70), but 10 years later it had increased by 5 years (from 70 to 74). Similar trends were observed in Slovenia (Jakovljevic and Laaser, 2015), South Korea, Iceland, Switzerland, Israel and elsewhere. In Thailand, LEAB had increased by 4 years (71–74) after it achieved UHC in 2002, compared to only 2 years (70–71) in the prior decade. China, Brazil, and the US all achieved strong economic growth, but life expectancy did not keep pace with those countries that achieved UHC. UHC can increase life expectancy in two ways. Firstly, disease mortality is reduced by minimizing risk factors through effective public health programs (Dye et al., 2013; World Health Organization, 2014b) and secondly, poverty is not a barrier to treatment if people get sick (Lagomarsino et al., 2012; Savedoff et al., 2012).

### Life expectancy and mortality

Life expectancy and all kinds of mortality are outcome indicators; however, they are inversely related to each other. If there is a high mortality among younger age groups, life expectancy will be shorter. Results from a demographic health survey in Bangladesh showed that high infant and child mortality rates result in lower values of life expectancy at birth than at older ages (Rabbi, 2013). Likewise, similar predictors affect life expectancy, infant, child and adult mortality. A study in Nigeria showed that education, household sanitation status and childhood care are associated with child mortality (Ezeh et al., 2015), and a study by Rutstein concluded that fertility, child care situation, socioeconomic status and access to health services were associated with infant and child mortality in developing countries (Rutstein, 2000). Put simply, higher child mortality obliviously reduced life expectancy.

Universal health coverage is not the same across countries with respect to levels of coverage, quality, and access to care; it is a single indicator with 3 components (see above). Nevertheless, as it is defined in this study, UHC has a strong influence on LEAB and HALE (World Health Organization, 1994). Our study highlighted that UHC has greater predictive power for long-term health outcomes than other components like education and economic growth rate (Ranabhat et al., 2017b). However, this study has some limitations, and generalization should be done with caution. Moreover, our data are drawn from different organizations, and the validity and reliability of the data remains a minor concern. Also, the sample size is not ideal from a statistical perspective even though we have used a large volume of data from all available countries. This study represents the first of its kind in terms of this type of analysis. It may not be sufficient to establish a fixed relationship between UHC and life expectancy without additional follow-up investigations. Further study, such as a longitudinal analysis, is recommended.

## Conclusion

There are different predictors for life expectancy. Child vaccination, sanitation and UHC all influence HALE and LEAB, with UHC having the greatest influence. Moreover, UHC reduced inequalities in HALE and LEAB. As such, the further adoption of UHC could play a key role in achieving improved global health outcomes. It is able to do this because it is a comprehensive health system approach that enables the provision of various effective health services that improve life expectancy at birth and healthy life expectancy. The current round of the global development agenda, the Sustainable Development Goal (SDGs), provides an excellent opportunity to design and implement specific programs to expand the ranks of countries that have achieved UHC.

## Availability of data and materials

The data are readily accessible and freely available from the websites of the respective organizations as described in Table 1.

## Author contributions

CR was chiefly responsible for the overall study, from formulating the research concept through to submission of the manuscript. C-BK made contributions to the research model and article composition. JA contributed to the overall article composition. M-BP and MJ reviewed the article and made further improvements.

### Conflict of interest statement

The authors declare that the research was conducted in the absence of any commercial or financial relationships that could be construed as a potential conflict of interest.

## Footnotes

^1^What is universal health coverage? [http://www.who.int/features/qa/universal_health_coverage/en/].

^2^Data [http://datacatalog.worldbank.org/?Topics=Health]

^3^Human Development Reports: Combined gross enrollment in education (both sex) % [http://hdr.undp.org/en/content/combined-gross-enrolment-education-both-sexes].

^4^Rising inequality between countries in adult length of life [http://voxeu.org/article/life-expectancy-around-world?quicktabs_tabbed_recent_articles block=0].

^5^Data: Life expectancy at birth, total (years) [http://data.worldbank.org/indicator/SP.DYN.LE00.IN].

## Abbreviations

LEAB: Life expectancy at birth
HALE: Healthy life expectancy
UHC: Universal health coverage
DPT: Diphtheria pertussis tetanus.
